# Association of liver biopsy pathology on outcome of patients undergoing heart transplantation

**DOI:** 10.1016/j.jhlto.2024.100187

**Published:** 2024-11-29

**Authors:** Lauren S. Eichenwald, Raffi Karagozian, Adam J. Eichenwald, John Morrissey, Saurav Kini, Ariella Stein, Amanda R. Vest

**Affiliations:** a Department of Gastroenterology, Dartmouth Hitchcock Medical Center, Lebanon, New Hampshire; b Department of Medicine, Tufts Medical Center, Boston, Massachusetts; c Department of Wildlife, Fisheries, and Conservation Biology, University of Maine, Orono, Maine; d Department of Cardiovascular Medicine, Cleveland Clinic, Cleveland, Ohio

**Keywords:** heart failure, liver fibrosis, cirrhosis, MELD-XI, heart transplant

## Abstract

**Background:**

Patients with advanced heart failure needing heart transplant commonly suffer liver dysfunction. However, there is limited data on the impact of liver fibrosis on outcomes for heart transplant (HT) candidates. We determine the relationship between liver fibrosis severity and mortality rates for HT patients.

**Methods:**

A retrospective cohort study of adults listed for HT who underwent a liver biopsy for evaluation of early or advanced liver fibrosis from August 12, 2004 to February 16, 2022. Trend analysis was performed using Cox proportional hazard model, controlling for MELD-XI. At-risk period starts at the time of waitlist; the end of the follow-up period was mortality on the waitlist, mortality post-HT, or administrative censoring at the end of the study.

**Results:**

There was no significant difference in the survival of patients with advanced fibrosis and early fibrosis over time (HR 1.54, CI 0.59–4.02, *p* = 0.5). Similarly, there was also no significant survival difference within groups who did (HR 0.78, CI = 0.26–2.33, *p* = 0.8) or did not (HR 1.00, CI 0.09–11.43, *p* = 0.9) receive transplants. However, most transplants were performed in patients with no or early fibrosis.

**Conclusion:**

There was no significant difference in the survival rates between HT candidates with and without advanced fibrosis on the waitlist and post-HT, challenging the notion that advanced fibrosis should be an absolute contraindication for HT. However, our findings are limited by the small sample size, retrospective design, and focus on patients already deemed suitable for transplantation. These limitations highlight the need for prospective studies involving broader patient populations, including those excluded from transplant candidacy due to severe fibrosis or cirrhosis. Future research should evaluate whether pre-transplant liver biopsy is necessary for all HT candidates or if clinical assessments can adequately stratify risk.

**Lay summary:**

This study found that the presence of advanced liver injury did not confer a difference in the waitlist and post heart transplant (HT) survival rates of patients on the HT transplant list. This finding suggests that patients listed for transplant may not need to undergo a liver biopsy as part of the transplant work up.

## Background

Patients with advanced heart failure who require heart transplant (HT) commonly have varying degrees of liver dysfunction, ranging from mild liver fibrosis to cirrhosis. This dysfunction is due to a combination of hepatic congestion from elevated hepatic venous pressure and impaired.^1^

The gold-standard for evaluating the severity of liver disease is liver biopsy, which is not infrequently performed prior to listing a patient for heart transplantation, particularly for patients with abnormal liver function tests, long-standing right ventricular failure, or imaging studies suggestive of liver cirrhosis.^2^

Current guidelines indicate that cirrhosis on liver biopsy may be a contraindication to heart transplantation in settings such as chronic viral hepatitis.^3^ However, the relationship between liver fibrosis and outcomes in heart transplant candidates remains unclear, as historical studies report conflicting findings. Hsu et al. found that heart transplantation in patients with liver cirrhosis is feasible and can achieve similar survival outcomes to those in patients without cirrhosis,^4^ suggesting that cirrhosis alone should not preclude consideration for heart transplantation. In contrast, Chokshi et al. found that higher preoperative model for end stage liver disease (MELD) scores were associated with significantly lower survival rates after heart transplantation.^5^ Other studies corroborate these findings in patients with advanced liver fibrosis.^6^, ^7^ These discrepancies underscore the complexity of balancing the risks and benefits of heart transplantation in patients with hepatic dysfunction. They also highlight the need for further research to determine when liver fibrosis severity should influence transplant candidacy. A better understanding of the relationship between liver fibrosis severity and outcomes in heart transplant candidates is crucial to inform clinical decision-making and optimize patient outcomes.

The purpose of this study was to further investigate the relationship between liver fibrosis severity and mortality rates for patients on the heart transplant waitlist and for patients in the post-heart transplant period. Given the historical literature suggesting that severe liver fibrosis or cirrhosis may be associated with a poorer post-heart transplant prognosis, it is important to note that our study population contained very few patients with advanced liver disease, as these patients would not have met the center-specific criteria for heart transplantation listing. Nonetheless, the implications of liver fibrosis, including mild to moderate grades, for outcomes in heart transplant candidates remains unclear due to limited data. Therefore, we hypothesized that early liver fibrosis in patients on the heart transplant waitlist would not significantly impact post-transplant survival. We further hypothesized that advanced liver fibrosis, within the context of this pre-selected transplant candidate population, would also not significantly affect survival due to the potential reversibility of congestive hepatopathy. This hypothesis is based on our understanding that liver injury in such patients is likely due to congestive hepatopathy caused by heart failure, and that the liver parenchyma changes will likely reverse after normal cardiac function is restored.

## Methods

We conducted a retrospective cohort study of patients from August 12, 2004 to February 03, 2023 at Tufts Medical Center in Boston, MA. The Tufts Medical Center/Tufts University Health Sciences Institutional Review Board evaluated this project and determined that it was exempt from the requirement for informed consent. Inclusion criteria were adults listed for HT who underwent a liver biopsy for evaluation of liver fibrosis. Clinical, laboratory and mortality data were obtained from the transplant center registry. All biopsies were performed via a transjugular approach. Forty-two patients met our criteria, with a mean age of 54.1 ± 12.5 years at the time of transplant (or at the time of death if the patient died while on the transplant list). Indications for heart transplantation were categorized as: (1) ischemic cardiomyopathy, (2) nonischemic cardiomyopathy, (3) hypertrophic cardiomyopathy, (4) chemotherapy associated cardiomyopathy, or (5) congenital heart disease.

The Ishak scoring system was adapted to stratify the patient cohort. The Ishak scoring system is widely used to stage fibrosis in the liver.^8^, ^9^ The system is based on the degrees of fibrosis and inflammation in the liver.^6^, ^10^ This system has been used in various studies to evaluate liver fibrosis in different populations, including patients with chronic hepatitis B or C, celiac disease, and those undergoing bariatric surgery.^6^, ^11^, ^12^ The Ishak scoring system has been shown to have good interobserver agreement and is a reliable tool for assessing liver histopathology.^13^ The degree of fibrosis was categorized as either early (Ishak score of 0–2) or advanced (score of 3–6). “Early fibrosis” is characterized by patients that had fibrosis expansion of portal areas with or without short fibrous septa or no fibrosis, while “advanced fibrosis” is characterized by patients with evidence of portal-to-portal bridging or cirrhosis.

To assess the relationship between liver fibrosis on post-transplant survival, we constructed a Cox proportional hazard model to perform trend analysis (R Studio, R Foundation, Austria). Fibrosis was treated as a binary categorical variable (early/advanced) to evaluate its association with survival outcomes. This model estimated the hazard of mortality during both the waitlist and post-transplant phases. The follow-up period began at waitlist entry and ended with 1 of 3 outcomes: mortality on the waitlist, mortality post-heart transplantation (HT), or administrative censoring at the study's end.

The purpose of the Cox model was to provide hazard ratios (HRs) and 95% confidence intervals (CIs) to quantify and adjust for the effect of fibrosis severity on survival outcomes. Additionally, the MELD-XI scoring system—a validated measure of liver function—was included as an adjustment covariate to control for baseline differences in liver function and minimize confounding effects. The proportional hazards assumption was verified using Schoenfeld residuals.

## Results

The study cohort (59.9 ± 11.3 years old) comprised individuals with diverse underlying diagnoses contributing to their heart conditions. Ischemic cardiomyopathy and nonischemic cardiomyopathy were equally prevalent, each diagnosed in sixteen participants. Additionally, hypertrophic cardiomyopathy was observed in 5 individuals, while chemotherapy-associated cardiomyopathy and congenital heart disease were each reported in two participants. Amyloidosis and arrhythmogenic right ventricular cardiomyopathy were less common, with only one case each identified in the cohort.

**Figure 1:**
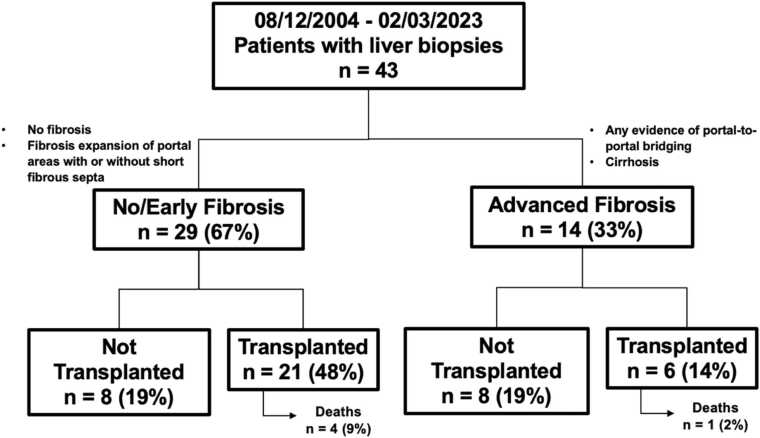
Number of no/early and advanced liver fibrosis patients who did and did not receive transplants.

Fourteen of the 43 patients (33%) had advanced fibrosis and 29 (67%) had no/early fibrosis (Figure 1). Two (5%) patients had cirrhosis. Eleven (26%) patients with no/early liver fibrosis died and 6 (14%) patients with advanced fibrosis died. Sixteen (37%) waitlisted patients did not receive heart transplants; 10 of these 16 patients died on the waitlist. Twenty-seven patients (63%) eventually underwent transplantation. Among the transplanted patients, the majority (21/27) had no/early fibrosis, while only 6 had advanced fibrosis, highlighting the limited representation of advanced fibrosis among the transplanted cohort. Nodular livers were present in 8 patients, normal livers were found in 13 patients, and 10 patients had echogenic livers without a nodular or coarsened appearance. Ascites was present in 12 patients. Unfortunately, the transition of our hospital's electronic medical record system to Epic resulted in the loss of radiographic and biopsy specimen gross information for 12 patients.

**Figure 2:**
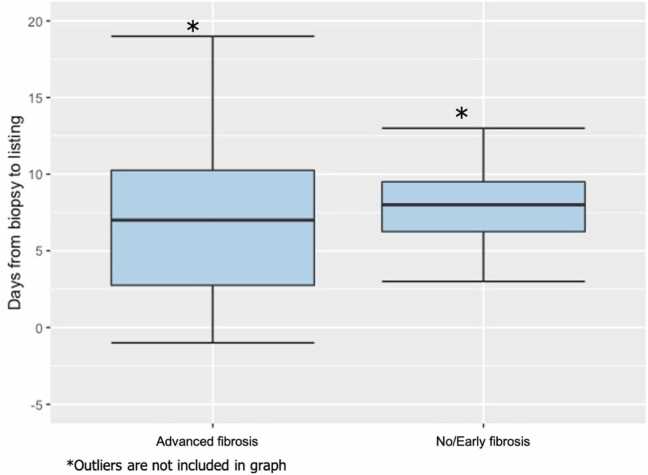
Box plot of number of days from time of liver biopsy to time of listing.

**Figure 3:**
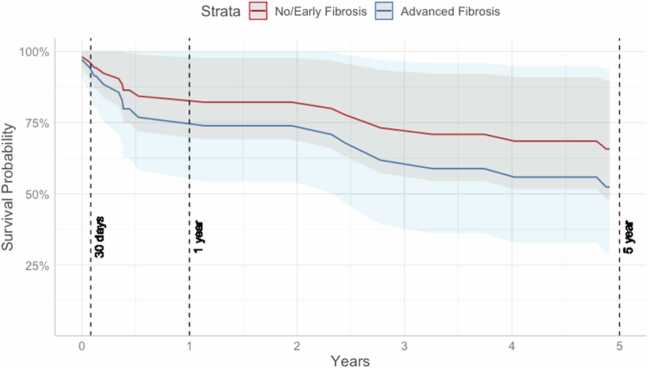
Predicted survival curve from time of listing for waitlisted patients, including those who did and did not receive transplants (model *p* = 0.5).

Most experts agree that a specimen with 11 portal tracts and about 3 cm long is adequate for evaluation.^14^ Most of the biopsy reports did not comment on the adequacy of the sample or the number of portal tracts (Supplemental Table 1), however, no report commented on the inadequacy of the sample, suggesting that the pathologist considered the specimen to be adequate for evaluation. We do note however, that there is a risk of sampling error given the geographic heterogeneity of fibrosis in a liver. In our cohort, the specimen size was reported as a range of sizes for each biopsy piece obtained; on average, 4 ± 1.5 specimens were obtained from each patient. The median size of each biopsy specimen was around 0.9 cm. Number of portal tracts was reported in only 5 patients. The average time from liver biopsy to listing was 124 days, with a median of 8 days (SD = 749 days; Figure 2). For both transplanted and non-transplanted patients, the predicted 5-year survival rate was 65.7% for those with no/early liver fibrosis and 52.4% for those with advanced liver fibrosis (Table 1, Figure 3). For all patients from time of listing, there was no significant difference in the survival of patients with advanced fibrosis compared to no/early fibrosis (HR 1.54, CI 0.59–4.02, *p* = 0.5).

**Table 1:** Predicted Survival Percentages for Transplanted Patients, Non-Transplanted Patients, and Both Groups Combined from Time of Listing

	Transplanted patients	Non-transplanted patients	Transplanted and non-transplanted patients^a^
	*n* = 27	*n* = 16	*n* = 43
Survival with No/Early Liver Fibrosis (%, CI)			
30 Days	100 (100−100)	86.6 (68.0−100)	96.2 (90.3−100)
1 Year	96.0 (87.5−100)	47.1 (22.4−99.3)	82.2 (69.1−97.8)
5 Years	87.7 (72.6−100)	13.7 (2.1−87.6)	65.7 (48.2−89.8)
Survival with Advanced Liver Fibrosis (%, CI)			
30 Days	100 (100−100)	89.4 (73.6−100)	94.2 (85.0−100)
1 Year	96.0 (84.7−100)	55.7 (30.0−100)	73.9 (54.3−100)
5 Years	87.8 (63.3−100)	21.3 (4.7−97.5)	52.4 (29.3−93.8)

CI, confidence interval.

a Analyzed with transplant as a time varying covariate.

**Figure 4:**
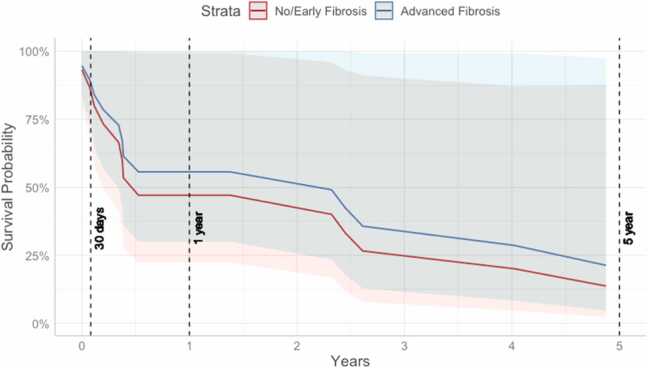
Predicted survival curve from time of listing for patients who did not receive transplants (model *p* = 0.8).

Among patients who did not receive heart transplants, the predicted survival rate at 5 years from the time of listing was 13.7% for patients with no/early liver fibrosis and 21.3% for patients with advanced liver fibrosis (Table 1, Figure 4; HR 0.78, CI = 0.26–2.33, *p* = 0.8). Patients who received heart transplants had a predicted survival of 87.7% at 5 years from the time of listing, and 87.8% had advanced fibrosis (Table 1; HR 1.00, CI 0.09–11.43, *p* = 0.9). Of those who received transplants, the predicted survival at 5 years from the time of transplant was 89.2% for patients with no/early liver fibrosis and 87.5% for patients with advanced liver fibrosis (Table 2; HR 1.1, CI = 0.10–13.50, *p* = 0.96). A subsequent survival analysis with age at list time as the dependent variable (controlling for MELD XI) also showed that differences in patient age did not influence survival over time (Hazard ratio = 1.041, 95% CI [0.990, 1.095], *p* = 0.117, Supplemental Table 2, Supplemental Figure 1).

**Table 2:** Predicted Survival Percentages for Transplanted Patients from Time of Transplant (Model *p* = 1.00)

Time from transplant	Survival with	Survival with
	No/Early liver fibrosis (%, CI)	Advanced liver fibrosis (%, CI)
	*n* = 21	*n* = 6
30 Days	96.5 (88.8−100)	95.9 (84.3−100)
1 Year	89.2 (75.3−100)	87.5 (62.8−100)
5 Years	89.2 (75.3−100)	87.5 (62.8−100)

Among those who died while awaiting transplantation, cardiovascular events, infections, and multiple organ failure were observed with similar frequencies. Cardiovascular events included cardiac arrest (2 cases) and ventricular failure (1 case), while infections, particularly bacterial septicemia (2 cases), and multiple organ failure (3 cases) were also notable contributors to mortality during the waitlist period. Additionally, respiratory failure (1 case) and unknown causes (2 cases) were reported. Among patients who underwent transplantation but succumbed thereafter, infections remained a significant concern. Bacterial pneumonia (1 case) and fungal infections, specifically aspergillosis (1 case), were identified as causes of mortality post-transplantation. As with the waitlist period, multiple organ failure (1 case) and respiratory failure (1 case) were observed. One death post-transplantation was categorized as unknown.

## Discussion

This study investigated the association between the severity of liver fibrosis and survival for heart transplant candidates. In our sample population, a greater number of patients with no or early liver fibrosis were transplanted than those with advanced fibrosis. This disparity is likely influenced by the pre-selection of transplant candidates, which favors individuals with milder liver fibrosis, as they are more likely to meet listing criteria and be considered viable for transplantation. The current consensus is that patients with no or early liver fibrosis are more likely to have better post-transplant survival rates.^2^ However, our survival analysis findings contrast with prior publications. We did not observe a significant difference in survival rates between heart transplant candidates with and without advanced fibrosis on the waitlist and post-transplant. This suggests that advanced fibrosis may not necessarily impact post-transplant survival in this specific population of patients. Instead, patients with advanced fibrosis could perhaps still achieve outcomes comparable to those without fibrosis, supporting the growing evidence that liver fibrosis alone should not preclude consideration for heart transplantation.^4^

Several factors may contribute to the comparable survival rates observed in patients with and without advanced liver fibrosis. Firstly, it is possible for the liver to regenerate itself once the underlying cardiac pathology is addressed, particularly in young congenital heart patients such as those with Fontan circulations.^15^ Indeed, the concept of congestive hepatopathy, in which liver dysfunction is primarily caused by hepatic congestion and impaired perfusion resulting from heart failure, may explain the reversibility of liver fibrosis in these patients. Additionally, advancements in perioperative management, including improved surgical techniques and post-transplant care, may have contributed to better outcomes regardless of liver fibrosis severity.^16^

Although these findings challenge the notion that advanced liver fibrosis should be an absolute contraindication for heart transplantation,^4^, ^17^, ^18^ it is important to acknowledge that these conclusions are derived from a small and retrospective dataset, which limits their generalizability and calls for cautious interpretation. Indeed, our limited sample size and absence of data on fibrosis etiology highlight the need for prospective studies to confirm these findings and assess their broader applicability. Furthermore, our analysis focuses exclusively on patients who were already deemed suitable for heart transplantation, inherently excluding the broader population of patients with more severe or advanced fibrosis who may not have been considered for transplantation. This selection bias limits the generalizability of our findings, as they may not fully represent outcomes for patients who present for transplant evaluation but fail to meet candidacy criteria. However, if our results are supported by further studies of the broader patient population, the pool of potential heart transplant candidates may be expanded by considering patients with advanced fibrosis who may have previously been deemed ineligible.

We note that the outcomes associated with combined heart-liver transplantation were not within the scope of our study. It is true that physicians might consider heart-liver transplant options for patients with advanced heart and liver disease. However, fewer than 500 cases of this procedure have been reported from the 1980s to 2023,^19^ underscoring its infrequency and complex nature. Despite its potential benefits for individuals with concurrent heart and liver conditions, standardized criteria for patient selection remain elusive, necessitating further investigation. Indeed, our center does not perform combined heart-liver transplants, precluding us from gathering firsthand data on this topic.

Additionally, we were unable to access data on patients' comorbidities, including the etiology of liver fibrosis, due to database limitations. While we assumed that liver fibrosis in this cohort was primarily a consequence of advanced heart failure, the potential contribution of unrelated etiologies cannot be excluded. As a result, our analysis lacks the granularity needed to disentangle the effects of liver fibrosis caused by heart failure from those caused by other conditions. This limitation complicates the ability to generalize our findings or fully discern the independent impact of fibrosis stemming from heart failure versus other causes. Furthermore, the lack of detailed pre- and post-transplant liver function data restricts our ability to directly evaluate the potential for liver fibrosis reversal and its subsequent effect on post-transplant outcomes. Future prospective studies incorporating comprehensive data on patients’ comorbidities, liver fibrosis etiology, and pre- and post-transplant liver function assessments are necessary to validate our findings, explore underlying mechanisms, and better understand the long-term implications of liver fibrosis on heart transplant outcomes.

In conclusion, while our study provides preliminary evidence that advanced liver fibrosis may not always be an absolute contraindication to heart transplantation, these findings must be interpreted with caution due to the limitations of our patient population. Future investigations should focus on prospective cohort studies that include broader patient populations and detailed clinical data, such as fibrosis etiology, liver function trends, and outcomes for non-listed patients, to confirm and extend these findings.

## Disclosure statement

The authors declare that they have no known financial interests or relationships that could have influenced this paper. The authors thank the clinical coordinators of the Tufts Heart Transplant Repository, particularly M. Wu for his assistance in retrieving the data from the repository.

## Footnotes

Appendix A Supplementary data associated with this article can be found in the online version at doi:10.1016/j.jhlto.2024.100187.
