# Supplementary material for: Association of liver biopsy pathology on outcome of patients undergoing heart transplantation
Source: JHLT Open. 2024 Nov 29;7:100187. doi: 10.1016/j.jhlto.2024.100187 (PMC11935397; doi:10.1016/j.jhlto.2024.100187)

**Supplemental Table 1. Liver biopsy specimen information and imaging findings at time of liver biopsy.**

| **Patient number** | **Liver fibrosis** | **Liver biopsy sample size** | **Number of portal tracts in biopsy specimen** | **Adequacy of biopsy specimen** | **Radiographic findings at time of liver biopsy** | **Presence of ascites at time of liver biopsy (Y/N)** |
| --- | --- | --- | --- | --- | --- | --- |
| **1** | Advanced Fibrosis | Four cylindrically shaped pieces of soft, tan-pink tissue. Ranging in size from 0.2cm in length to 0.7cm in length and having a diameter of less than 0.1cm | Not reported | Not reported | "Mild fatty liver infiltration" | N |
| **2** | Advanced Fibrosis | Four cylindrical tan-pink tissues ranging from 0.8cm-1.8cm in length and each 0.1cm in diameter. 5.1cm in aggregate | Not reported | Not reported | "Focal hepatic calcification, likely calcified granulomata. Otherwise normal liver parenchyma" | Y |
| **3** | No/Early Fibrosis | Multiple cylindrically shaped pieces of soft tan-brown tissue ranging in size from 0.5-0.9cm in greatest dimension | Not reported | Not reported | "Heterogeneity of the liver, which is felt to me most consistent w venous congestion secondary to the patient's cardiac disease" | Y |
| **4** | No/Early Fibrosis | Three cylindrical tan brown tissues ranging from 1-1.5cm in length and each 0.1cm in diameter | Not reported | Not reported | "The liver is slightly heterogeneous in echotexture, and is slightly lobulated in contour. The liver has a somewhat nodular contour of uncertain significance" | Y |
| **5** | No/Early Fibrosis | Two cylindrically shaped pieces of rubbery, tan brown tissue ranging in size from 0.6 up to 1.4cm in length and having a diameter of 0.2cm | Not reported | Not reported | "Homogenous liver parenchymal echotexture" | N |
| **6** | No/Early Fibrosis | Five cores of soft pale brown tissue measuring 0.5-1.3cm in length by less than 0.1cm in diameter | Not reported | Not reported | "Mild coarsening and slight increased echogenicity of the liver. Early hepatic steatosis or nonspecific hepatitis including cirrhosis and fibrosis can lead to this appearance" | N |
| **7** | No/Early Fibrosis | Four cylindrically shaped pieces of rubbery tan brown tissue ranging in size from 0.3-0.8cm in length and having diameter of 0.1cm | Not reported | Not reported | "Normal liver parenchyma. Nonspecific hyperechoic mass w irregular boarders, 3.2x1.8x3.4cm (could be focal fatty infiltration, Transjugular hemangioma, tumor)" | N |
| **8** | No/Early Fibrosis | Three cylindrically shaped pieces of rubbery tan brown tissue ranging in size from 0.4-0.8 cm in length and having a diameter of 0.1cm | Not reported | Not reported | "Homogenous parenchymal echo texture without focal lesion. However, prominence of portal triads noted which could be the result of congestive failure" | N |
| **9** | No/Early Fibrosis | Six cylindrical fragments of soft tan tissue, measuring 0.1cm in average diameter and from 0.6-2.0 cm long | Not reported | Not reported | "Liver is normal in echotexture and contour" | N |
| **10** | No/Early Fibrosis | Greater than 5 delicate pink tan cylindrical tissues (1.5cmx0.4cmx0.1cm in aggregate) | "This large core biopsy consists of several portal tracts for analysis" | Not reported | "Liver is normal in echotexture and contour" | N |
| **11** | No/Early Fibrosis | Three cylindrically shaped pieces of rubbery, tan yellow tissue ranging in size from 0.3cm in length up to 1.2cm in length and having diameter 0.1cm | "At least 5 portal fields are available for evaluation" | Not reported | "The liver is normal without focal abnormalities. There is enlargement of hepatic veins indicating congestion" | Y |
| **12** | Advanced Fibrosis | Three cylindrically shaped pieces of rubbery, than brown tissue each measuring approximately 1.7cm in length and having a diameter of less than 0.1cm | Not reported | Not reported | "The liver is homogenous in echotexture without evidence of focal lesion. Portions of the liver surface are nodular in contour" | Y |
| **13** | Advanced Fibrosis | The biopsy is composed by multiple fragments aggregating to 4.8cm in total length. | "Approximately 13 partial or complete portal triads are present for review" | Not reported | "The liver is slightly nodular in contour" | Y |
| **14** | No/Early Fibrosis | Multiple cores each approximately 2.0cm | "13 portal tracts are available for evaluation" | Not reported | "Diffusely hypoattenuating consistent with diffuse fatty infiltration" | N |
| **15** | Advanced Fibrosis | n/a | n/a | n/a | n/a | n/a |
| **16** | No/Early Fibrosis | n/a | n/a | n/a | n/a | n/a |
| **17** | No/Early Fibrosis | Multiple cores each approximately 2.5cm | Not reported | Not reported | "Hepatomegaly, heterogenous & coarsened in echotexture" | Y |
| **18** | No/Early Fibrosis | Three cores ranging from 1.2cm -1.8cm | Not reported | Not reported | "Minimal heterogeneous echotexture" | N |
| **19** | No/Early Fibrosis | Four cores ranging from 0.3cm-1.1cm | Not reported | Not reported | "Normal echogenicity" | N |
| **20** | No/Early Fibrosis | Multiple cores ranging from 0.8cm-1.4cm | Not reported | Not reported | "Normal echotexture and contour" | N |
| **21** | No/Early Fibrosis | Multiple cores each approximately 4cm | Not reported | Not reported | "Diffusely echogenic with a coarsened echotexture consistent with fatty infiltration" | Y |
| **22** | Advanced Fibrosis | Eight cores ranging from 0.3cm - 1.7cm | Not reported | Not reported | "Echogenic and mildly coarsened liver" | N |
| **23** | No/Early Fibrosis | n/a | n/a |  | n/a | n/a |
| **24** | No/Early Fibrosis | Four cores each approximately 1.8 cm | Not reported | Not reported | "Normal echotexture and contour" | N |
| **25** | No/Early Fibrosis | Six cores ranging from 0.3cm to 1.5cm | Not reported | Not reported | "Minimally increased echogenicity compatible with hepatic steatosis" | Y |
| **26** | No/Early Fibrosis | Two cores each approximately 3.1cm | Not reported | Not reported | "Heterogenous echogenicity, Nodular" | Y |
| **27** | No/Early Fibrosis | n/a | n/a | n/a | n/a | n/a |
| **28** | Advanced Fibrosis | n/a | n/a | n/a | n/a | n/a |
| **29** | No/Early Fibrosis | Three cylindrical tissues, ranging from 1.9-2.3cm in length, each 0.1cm in diameter. 4.2cm in aggregate | "Portal tracts are adequate for evaluation" | Not reported | "Liver appears unremarkable" | N |
| **30** | No/Early Fibrosis | n/a | n/a | n/a | n/a | n/a |
| **31** | Advanced Fibrosis | Two cylindrical fragments, 1.2cm and 0.8cm in length and 0.2cm in diameter | "At least 4 portal tracts" | Not reported | "Nodular liver" | Y |
| **32** | No/Early Fibrosis | Six cylindrical tissues, ranging in size from 0.5-1.1cm in length and 0.1cm in diameter | Not reported | Not reported | "Liver contour and echotexture is normal" | N |
| **33** | Advanced Fibrosis | Four cylindrical tissues, ranging from 0.6 cm to 1.8cm in length and each less than 0.1cm in diameter | Not reported | Not reported | "Liver echotexture is mildly increased, suggestive of steatosis, no focal hepatic lesions" | N |
| **34** | Advanced Fibrosis | n/a | n/a | n/a | n/a | n/a |
| **35** | Advanced Fibrosis | Four cylindrical tissues, ranging from 0.4-1.3 cm in length and 0.1cm in diameter | Not reported | Not reported | "Diffusely heterogeneous and nodular in contour, compatible with cirrhosis. Increased attenuation of the liver" | N |
| **36** | Advanced Fibrosis | n/a | n/a | n/a | n/a | n/a |
| **37** | Advanced Fibrosis | n/a | n/a | n/a | n/a | n/a |
| **38** | No/Early Fibrosis | n/a | n/a | n/a | n/a | n/a |
| **39** | No/Early Fibrosis | n/a | n/a | n/a | n/a | n/a |
| **40** | No/Early Fibrosis | n/a | n/a | n/a | n/a | n/a |
| **41** | No/Early Fibrosis | Three cylindrical fragments, ranging 1.3cm to 1.6cm in length, each less than 0.1cm in diameter | Not reported | Not reported | "Liver unremarkable" | N |
| **42** | Advanced Fibrosis | Three cylindrical fragments, ranging from 1.5-1.7cm in length, each less than 0.1 cm in diameter | "4 portal tracts" | Not reported | "Surface contour of the liver appears nodular, consistent with cirrhosis" | Y |
| **43** | No/Early Fibrosis | Six cylindrical fragments, ranging from 0.1-0.2cm in diameter, and 0.1cm to 1.8cm in length | Not reported | Not reported | "Liver is normal in echotexture and contour" | N |

**Supplemental Table 2. Predicted survival percentages by age for waitlisted patients, including those who did and did not receive transplants.**


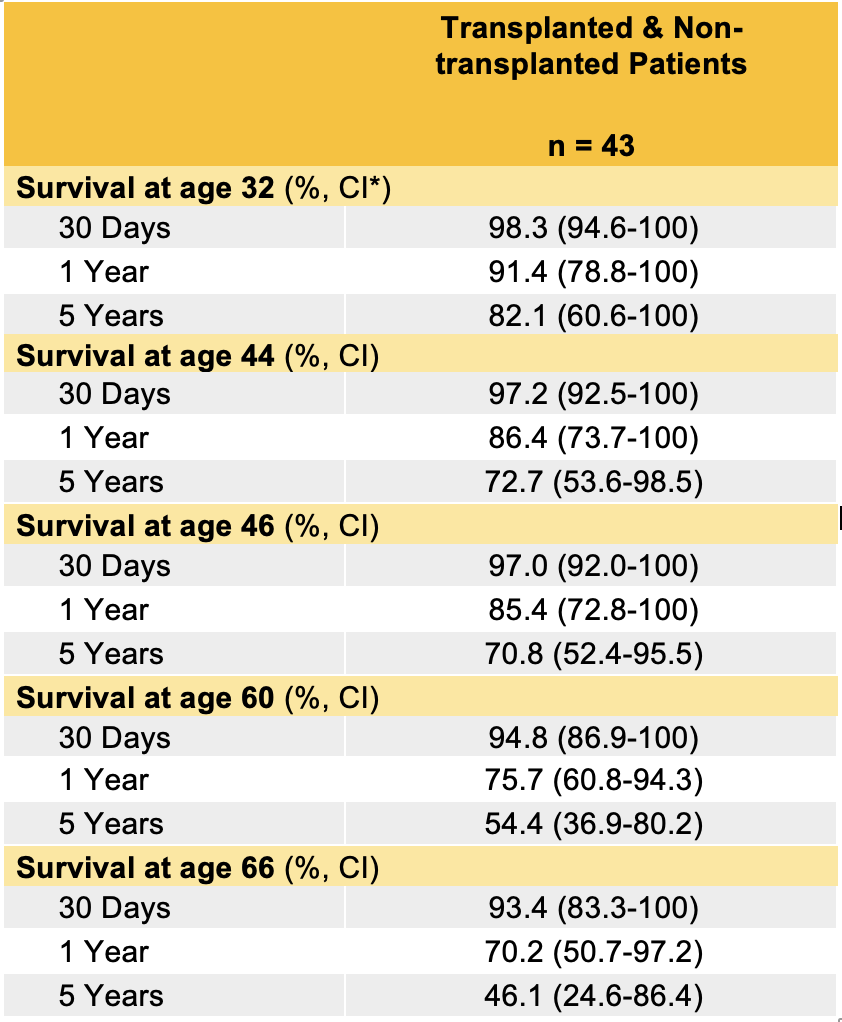


**Supplemental Figure 1: Predicted survival curve from time of listing for waitlisted patients, including those who did and did not receive transplants by age (model p = 0.1)**


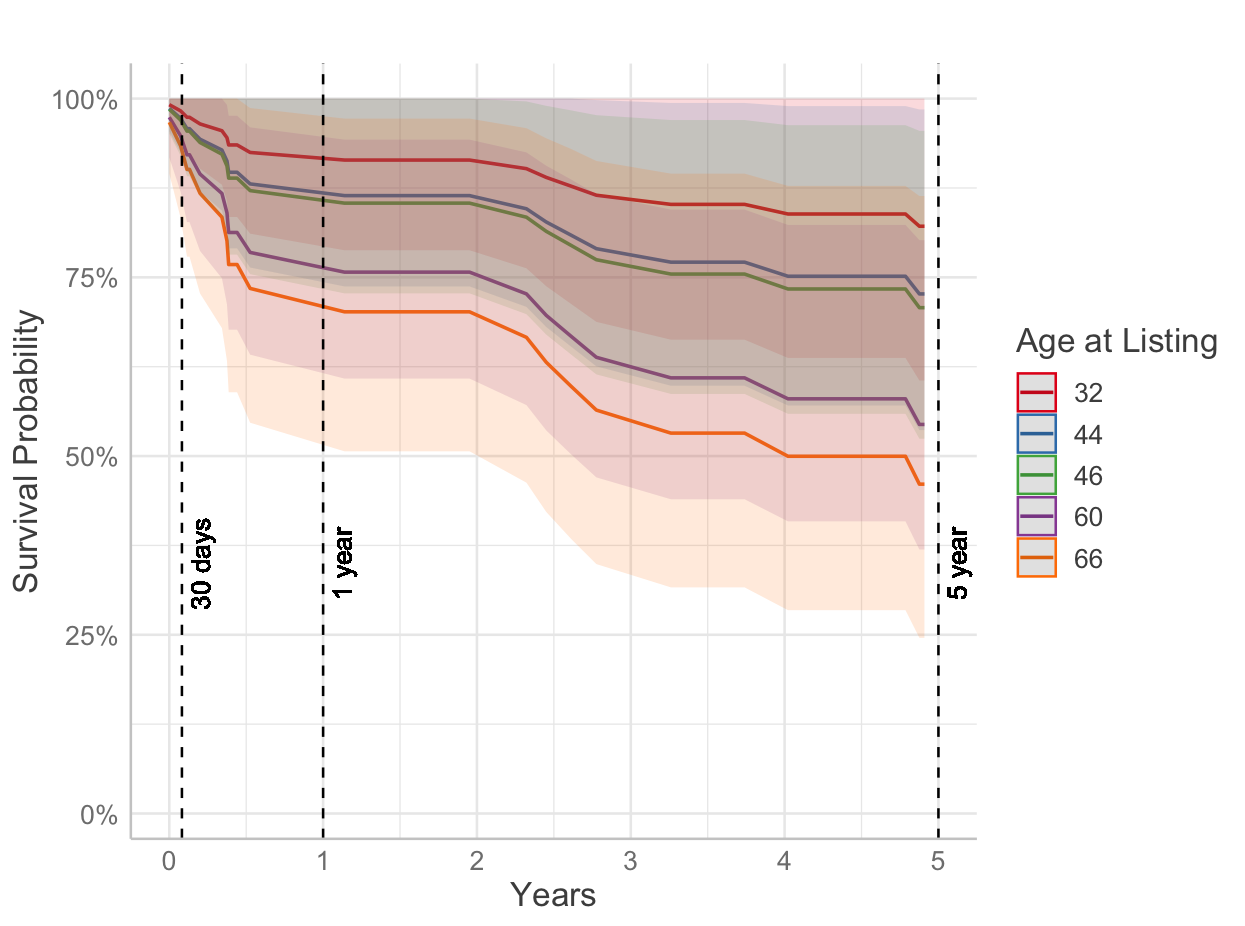

Supplement: Supplementary file 1 — Supplementary material [file mmc1.docx]
